# Development and validation of deep learning classifiers to detect Epstein-Barr virus and microsatellite instability status in gastric cancer: a retrospective multicentre cohort study

**DOI:** 10.1016/S2589-7500(21)00133-3

**Published:** 2021-08-17

**Authors:** Hannah Sophie Muti, Lara Rosaline Heij, Gisela Keller, Meike Kohlruss, Rupert Langer, Bastian Dislich, Jae-Ho Cheong, Young-Woo Kim, Hyunki Kim, Myeong-Cherl Kook, David Cunningham, William H Allum, Ruth E Langley, Matthew G Nankivell, Philip Quirke, Jeremy D Hayden, Nicholas P West, Andrew J Irvine, Takaki Yoshikawa, Takashi Oshima, Ralf Huss, Bianca Grosser, Franco Roviello, Alessia d'Ignazio, Alexander Quaas, Hakan Alakus, Xiuxiang Tan, Alexander T Pearson, Tom Luedde, Matthias P Ebert, Dirk Jäger, Christian Trautwein, Nadine Therese Gaisa, Heike I Grabsch, Jakob Nikolas Kather

**Affiliations:** a Department of Medicine III, University Hospital RWTH Aachen, Aachen, Germany; b Department of Surgery and Transplantation, University Hospital RWTH Aachen, Aachen, Germany; c Institute of Pathology, University Hospital RWTH Aachen, Aachen, Germany; d Institute of Pathology, TUM School of Medicine, Technical University of Munich, Munich, Germany; e Institute of Pathology, Inselspital, University of Bern, Switzerland; f Institute of Pathology and Molecular Pathology, Kepler University Hospital, Johannes Kepler University Linz, Linz, Austria; g Department of Surgery, Yonsei University Health System, Yonsei University College of Medicine, Seoul, South Korea; h Department of Pathology, National Cancer Center, Goyang, South Korea; i Center for Gastric Cancer, National Cancer Center, Goyang, South Korea; j Department of Pathology, Yonsei University College of Medicine, Seoul, South Korea; k Department of Medicine, Gastrointestinal and Lymphoma Units, The Royal Marsden NHS Foundation Trust, London, UK; a Department of Surgery, Royal Marsden Hospital, London, UK; m Medical Research Council Clinical Trials Unit, University College London, London, UK; n Pathology and Data Analytics, Leeds Institute of Medical Research at St James's, University of Leeds, Leeds, UK; o Department of Oesophago-Gastric Surgery, St James's University Hospital, Leeds, UK; p Department of Gastric Surgery, National Cancer Center Hospital, Tokyo, Japan; q Department of Gastrointestinal Surgery, Kanagawa Cancer Center, Yokohama, Japan; r Institute of Pathology and Molecular Diagnostics, University Hospital Augsburg, Augsburg, Germany; s Department of Medicine, Surgery and Neuroscience, Unit of General Surgery and Surgical Oncology, University of Siena, Italy; t Institute of Pathology, University Hospital Cologne, Cologne, Germany; u Department of General, Visceral, Cancer and Transplantation Surgery, University Hospital Cologne, Cologne, Germany; v Department of Medicine, University of Chicago Medicine, Chicago, IL, USA; w Department of Gastroenterology, Hepatology and Infectious Diseases, University Hospital Duesseldorf, Düsseldorf, Germany; x Department of Medicine II, Mannheim Institute for Innate Immunoscience and Clinical Cooperation Unit Healthy Metabolism, Center of Preventive Medicine and Digital Health, Medical Faculty Mannheim, Heidelberg University, Mannheim, Germany; y Department of Medical Oncology, National Center for Tumor Diseases, University Hospital Heidelberg, Heidelberg, Germany; z Department of Pathology, GROW School for Oncology and Developmental Biology, Maastricht University Medical Center+, Maastricht, Netherlands

## Abstract

**Background:**

Response to immunotherapy in gastric cancer is associated with microsatellite instability (or mismatch repair deficiency) and Epstein-Barr virus (EBV) positivity. We therefore aimed to develop and validate deep learning-based classifiers to detect microsatellite instability and EBV status from routine histology slides.

**Methods:**

In this retrospective, multicentre study, we collected tissue samples from ten cohorts of patients with gastric cancer from seven countries (South Korea, Switzerland, Japan, Italy, Germany, the UK and the USA). We trained a deep learning-based classifier to detect microsatellite instability and EBV positivity from digitised, haematoxylin and eosin stained resection slides without annotating tumour containing regions. The performance of the classifier was assessed by within-cohort cross-validation in all ten cohorts and by external validation, for which we split the cohorts into a five-cohort training dataset and a five-cohort test dataset. We measured the area under the receiver operating curve (AUROC) for detection of microsatellite instability and EBV status. Microsatellite instability and EBV status were determined to be detectable if the lower bound of the 95% CI for the AUROC was above 0·5.

**Findings:**

Across the ten cohorts, our analysis included 2823 patients with known microsatellite instability status and 2685 patients with known EBV status. In the within-cohort cross-validation, the deep learning-based classifier could detect microsatellite instability status in nine of ten cohorts, with AUROCs ranging from 0·597 (95% CI 0·522–0·737) to 0·836 (0·795–0·880) and EBV status in five of eight cohorts, with AUROCs ranging from 0·819 (0·752–0·841) to 0·897 (0·513–0·966). Training a classifier on the pooled training dataset and testing it on the five remaining cohorts resulted in high classification performance with AUROCs ranging from 0·723 (95% CI 0·676–0·794) to 0·863 (0·747–0·969) for detection of microsatellite instability and from 0·672 (0·403–0·989) to 0·859 (0·823–0·919) for detection of EBV status.

**Interpretation:**

Classifiers became increasingly robust when trained on pooled cohorts. After prospective validation, this deep learning-based tissue classification system could be used as an inexpensive predictive biomarker for immunotherapy in gastric cancer.

**Funding:**

German Cancer Aid and German Federal Ministry of Health.

## Introduction

Gastric cancer is among the most common and lethal cancer types worldwide.^1^, ^2^ Although the development of new treatment strategies had stalled for decades, the field was reinvigorated by the emergence of immunotherapy in the past decade.^1^ Among all genetic subclasses of gastric cancer, tumours with microsatellite instability (or mismatch repair deficiency) are associated with an improved response to immunotherapy. Correspondingly, immune checkpoint inhibitors are approved by the US Food and Drug Administration for metastatic gastric cancers with microsatellite instability.^3^ Additionally, microsatellite instability is a prognostic biomarker associated with an improved long-term prognosis.^4^, ^5^ Another driving mechanism for approximately 5% of gastric cancers is Epstein-Barr virus (EBV); these cancers are characterised by a vigorous immune response^6^ and potential susceptibility to immunotherapy.^3^ Conversely, EBV-negative gastric cancers and those with microsatellite stability have shown favourable outcomes after adjuvant chemotherapy.^7^ Microsatellite instability and EBV positivity are almost mutually exclusive, making these biomarkers complementary predictors for response to immunotherapy.^8^ Microsatellite instability is routinely assessed via PCR or immunohistochemistry.^9^ For EBV, the gold standard test is in-situ hybridisation to detect EBV-encoded RNA transcripts.^10^ However, these tests are not ubiquitously done even in health-care systems with plentiful resources.

**Research in context**

**Evidence before this study**

Gastric cancer is one of the most lethal types of cancer across all countries and ethnicities. The Cancer Genome Atlas (TCGA) project divided gastric cancer into four molecular subtypes, one of which is microsatellite instable and one of which is Epstein-Barr virus (EBV) positive gastric cancer. Deep learning, a method within artificial intelligence (AI), has successfully detected molecular alterations directly from histopathology slides in previous studies. We searched PubMed, MEDLINE, Google Scholar, and conference abstracts from IEEE Symposia on Jan 13–17, 2020, for literature published since database inception, with no language restrictions, on deep learning-based molecular detection in gastric cancer using the keywords “digital pathology”, “deep learning”, and “histopathology” in combination with “EBV”, “Epstein Barr virus”, “prediction”, “detection” or “molecular detection”, “microsatellite instability”, “gastric cancer”, and “gastric adenocarcinoma”. Although some publications reported tumour detection in gastric cancer and one publication reported the detection of microsatellite instability or EBV status in the TCGA cohort, we did not identify any large scale validation studies describing deep learning-based detection of microsatellite instability and EBV status in gastric cancer. We repeated our literature search on July 2, 2021, and found one additional publication reporting the detection of microsatellite instability from routine histology using deep learning, but large scale systematic validation studies are still unavailable.

**Added value of this study**

We assembled a multi-institutional dataset comprising more than 2500 patients with gastric cancer from ten clinical cohorts from several countries worldwide. We show that deep learning-based prediction of microsatellite instability and EBV status from haematoxylin and eosin-stained histopathological samples is feasible. We compared the performance of our deep learning-based classifiers on various sample types including whole-slide images, full tumour annotations, virtual biopsies, and tissue microarrays and found that manual tumour annotations are not needed for deep learning-based detection of microsatellite instability and EBV status. Additionally, we found that classifier performance increased substantially with pooling of cohorts and is largely independent of clinicopathological characteristics.

**Implications of all the available evidence**

In the future, AI could be used to screen patients with gastric cancer for the presence of clinically relevant genetic alterations. This process could reduce the number of molecular tests required and enable universal screening potentially even in low-resource health-care systems. If deep learning systems were used to identify molecular alterations globally, pathologists and clinicians could make faster clinical decisions and offer therapeutic approaches tailored to the molecular profile of the individual patient. Furthermore, the deep learning pipeline presented in this study can be applied to other disease contexts, parameters, or populations of interest. Our strategies to improve detection accuracy in previously problematic cohorts could also inform study conceptualisation approaches for other researchers.

The number of molecular tests required could be reduced by detection of genetic abnormalities directly from routine histology.^11^ Deep learning, an artificial intelligence (AI) technology, is ideal for extracting subtle information from complex data.^12^ Several studies have shown that deep learning algorithms can detect the presence of molecular alterations from routine histological data.^13^, ^14^, ^15^ In particular, deep learning can be used to detect microsatellite instability in colorectal,^11^, ^15^, ^16^, ^17^, ^18^, ^19^ endometrial,^11^, ^20^ and gastric cancer.^11^, ^21^, ^22^ To our knowledge, deep learning-based detection of EBV in gastric cancer has not been investigated to date. Clinical adoption of deep learning-based classification requires evidence from multicentre studies and large-scale evaluation, but no such studies have been done for any molecular biomarker in gastric cancer. To address this unmet need, we collected data from ten gastric cancer cohorts from several countries, and developed and assessed deep learning-based classifiers to detect microsatellite instability and EBV status directly from haematoxylin and eosin-stained histological slides.

## Methods

### Study design and patient cohorts

In this retrospective, multicentre cohort study, we collected digitised histological slides from formalin-fixed paraffin-embedded gastric cancer resection samples with matched microsatellite instability and EBV status from ten cohorts of patients with gastric cancer. Samples from the ten cohorts were as follows: samples from the pathology archives of Inselspital, University of Bern (Bern, Switzerland—ie, the BERN cohort);^23^ samples from the CLASSIC trial from participating study centres in South Korea (ie, the CLASSIC cohort);^24^ samples from the Medical Research Council Adjuvant Gastric Infusional Chemotherapy (MAGIC) trial from participating study centres in the UK (ie, the MAGIC cohort);^25^ samples from the Leeds Teaching Hospitals National Health Service Trust (Leeds, UK—ie, the LEEDS cohort); samples from the Kanagawa Cancer Center Hospital (Yokohama, Japan—ie, the KCCH cohort);^26^ samples from the pathology archive at the University Hospital Augsburg (Augsburg, Germany—ie, the AUGSB cohort); samples from the University of Siena (Siena, Italy—ie, the ITALIAN cohort);^27^ samples from the pathology archive at University of Cologne (Cologne, Germany—ie, the KOELN cohort);^28^ samples from the Institute of Pathology at the Technical University Munich (Munich, Germany—ie, the TUM cohort);^4^ and samples (diagnostic slides) originate from the The Cancer Genome Atlas (TCGA) project and are derived from the National Institute of Health Genomic Data Commons portal (the TCGA cohort).^8^, ^29^

This study was done in accordance with the Declaration of Helsinki and complies with the STARD reporting guidelines (appendix pp 2–4).^30^ This study was approved by the ethics board at RWTH Aachen University Hospital and the collection of patient samples in each cohort was approved by the ethics board at each institution.

### Deep learning

We processed whole-slide images (appendix p 11) from patients with known microsatellite instability status and patients with known EBV status from multiple countries, using one slide per patient. In the ITALIAN cohort, which consisted only of tissue microarrays, all available core samples per patient were used. We then trained and assessed deep neural networks as follows.

First, we separately trained and validated deep learning-based detectors for microsatellite instability and EBV status within each cohort in a three-fold cross-validated design, splitting each cohort into three datasets and rotating to use every dataset for validation once. The resulting prediction scores were used for a subgroup analysis to assess performance using the following clinicopathological strata: sex, Laurén subtype of gastric cancer (intestinal, non-intestinal or diffuse, mixed), Union for International Cancer Control (UICC) stage (stage I, II, III and IV), and grade of differentiation (1, 2, or 3–4).

Second, we externally validated our classification approach. We created a pooled training dataset from the five largest cohorts: BERN, CLASSIC, MAGIC, LEEDS, and TCGA. We started by training and assessing a deep learning-based classifier within this training dataset using within-cohort three-fold cross-validation, yielding one cross-cohort prediction area under the receiver operator curve (AUROC). A new classifier was then trained on the pooled training dataset and separately validated on each of the remaining cohorts (KCCH, AUGSB, ITALIAN, KOELN, and TUM). KOELN was excluded from validation of the EBV detection classifier because only two patients in this cohort were EBV positive.

Third, we did a three-way classification. Exploiting the almost perfect exclusiveness of microsatellite instability and EBV positivity, a three-way classifier was trained to distinguish between EBV-positive, microsatellite instable, and double-negative tumours (ie, negative for both EBV and microsatellite instability), and was assessed in a within-cohort cross-validation design. The MAGIC and KOELN cohorts, where EBV status was not available in a sufficiently large number of patients, and three patients with overlapping positive microsatellite instability and EBV status (two from the CLASSIC cohort and one from the LEEDS cohort) were excluded.

Fourth, we compared our baseline approach (ie, no annotations) with manual tumour-only annotations and virtual biopsy annotations. Tumours were annotated by a trained observer (HSM) and reviewed by pathologists (LRH, HIG, NTG) as previously described.^16^, ^31^ For virtual biopsy annotations, we created a 2 mm wide annotation of the tumour and adjacent healthy tissue facing the gastric luminal surface, simulating the tissue of an endoscopic biopsy sample.^32^ We deployed the classifier from our external validation step on tumour-only and virtual biopsy annotations for all eligible cohorts (TUM, KCCH, and AUGSB) to compare classifier performance in specified regions instead of using a whole-slide image. These three cohorts were used to directly compare our baseline external validation approach with the performance of tumour-only and virtual biopsy regions. Our other two validation cohorts were excluded from this experiment because of the low number of EBV positive cases (KOELN) or availability of tissue microarray cores only (ITALIAN).

Finally, we analysed classifier performance stratified by tumour-to-total tissue ratio of each slide. Based on tumour annotations, patients were stratified by the ratio between tumour area and total tissue area into low (0–0·33), medium (0·34–0·66), or high (>0·66).

### Image processing and statistical analysis

Histological slides were selected and digitised at each institution using Aperio (Leica Biosystems, Wetzlar, Germany), Hamamatsu (Hamamatsu Photonics, Hamamatsu-city, Japan), Ventana (Roche, Basel, Switzerland), or 3D Histech (3DHISTECH, Budapest, Hungary) digital slide scanners. All samples were surgical resections except for those from the ITALIAN cohort, which consisted of tissue microarrays. All data were preprocessed according to a prespecified protocol.^31^ Briefly, whole-slide images were tessellated into square image patches (tiles) with an edge length of 256 μm equivalent to 512 × 512 pixels, corresponding to a magnification of 0·5 μm per pixel, removing tissue-less background by discarding tiles with a median brightness above 220/255 (dimensionless factor) with QuPath (version 0.1.2).^33^ All tiles were colour-normalised using the Macenko method.^34^ All experiments were done on servers with NVIDIA (Santa Clara, CA, USA) RTX Titan or RTX 6000 graphics processing units using Matlab R2020a (Mathworks, Natick, MA, USA). Before training, tiles were randomly under-sampled to achieve class balance between microsatellite stability and instability or between EBV positivity and negativity—ie, if the less abundant class had N tiles, only N tiles were randomly chosen from the more abundant class. This approach balanced the training dataset without affecting the number of patients.^14^ The maximum number of tiles per patient was limited to 2000. We used a shufflenet model with an input size of 512 × 512 × 3 pixels, which was pretrained on ImageNet^35^ and retrained on each training dataset via transfer learning. The penultimate layer was replaced by a fully convolutional layer and the output layer was replaced with one neuron per output class. Training hyperparameters are listed in the appendix (p 5). For deployment, a categorical prediction was generated for each tile (tile-level hard prediction). For a given patient, the fraction of all tiles predicted to be of the target (microsatellite instability or EBV positive) was used as a patient-level prediction score, which can be converted to a patient-level hard prediction at different operating thresholds in a receiver operating curve (ROC) analysis.

For within-cohort experiments, stratified patient-level three-fold cross-validation was used. No data from the same patient were ever present in the training dataset and in the test dataset in any experiment. We report all results as patient-level AUROCs, with pointwise 95% CIs calculated in a ten-fold bootstrapping experiment. A classification was regarded as successful if the lower bound of the 95% CI was above 0·5. Within-cohort cross-validation and external validation were repeated with 1000-fold bootstrapping. The influence of the number of folds in the cross-validation was systematically assessed for this experiment in the BERN cohort. The BERN cohort was chosen for this analysis because it was representative of all the cohorts with respect to cohort characteristics. For subgroup-dependent ROC analyses, all patient-level predictions in a particular subgroup (eg, only female patients) were used. Subgroup analyses were only done in cohorts with sufficient patient-level data for the particular subgroups. We assessed statistical significance using a two-tailed unpaired Student's *t* test on the patient-level scores with p values of less than 0·05 indicating statistical significance.

All image processing steps were predefined and were not tuned specifically to the datasets in this study. All procedures followed an established protocol used in previous studies.^14^, ^16^

### Role of the funding source

The funders of the study had no role in the study design, data collection, data analysis, data interpretation, or writing of the report.

## Results

Across the ten cohorts, our analysis included 2823 patients with known microsatellite instability status and 2685 patients with known EBV status. Study profiles for all cohorts are shown in the appendix (p 10). Clinical and demographic characteristics of patients in each cohort are shown in table 1. Across all cohorts, the majority of patients were male and were diagnosed with UICC stage II or III (locally advanced resectable disease). Patients in the KCCH and CLASSIC cohorts originated from Asia, the rest of the patients were from Europe or the USA. Most tumours were poorly differentiated. Mutation frequency, presurgical or postsurgical pretreatment, microsatellite instability detection method, and slide scanner manufacturer varied between cohorts.

**Table 1:** Clinicopathological characteristics of the cohorts

			**BERN (N=418)**	**CLASSIC (N=612)**	**MAGIC (N=263)**	**LEEDS (N=903)**	**TCGA (N=443)**	Pooled cohort (N=2639)*	**KCCH (N=252)**	**AUGSB (N=181)**	**ITALIAN (N=398)**	**KOELN (N=372)**	**TUM (N=286)**
Country of origin			Switzerland	South Korea	UK	UK	USA	..	Japan	Germany	Italy	Germany	Germany
Patients included in this study			296	612	253	319	334	NA	252	181	366	84†	286
	Microsatellite instability		42 (14%)	32 (5%)	17 (7%)	33 (10%)	58 (17%)	182 (7%)	22 (9%)	16 (9%)	68 (19%)	4 (5%)†	34 (12%)
	Microsatellite stability		252 (85%)	535 (87%)	236 (93%)	282 (88%)	275 (82%)	1580 (60%)	213 (85%)	165 (91%)	218 (60%)	80 (95%)†	241 (84%)
	EBV positive		8 (3%)	41 (7%)	NA	14 (4%)	27 (8%)	90 (3%)	11 (4%)	3 (2%)	7 (2%)	2 (2%)†	8 (3%)
	EBV negative		288 (97%)	559 (91%)	NA	299 (94%)	306 (92%)	1452 (55%)	223 (88%)	178 (98%)	357 (98%)	87 (103%)†	267 (93%)
	Sample type		Whole slide	Whole slide	Whole slide	Whole slide	Whole slide	NA	Whole slide	Whole slide	Tissue microarray	Whole slide	Whole slide
	Age, years		68·9 (61·0–78·3)	57·0 (NA)	62·0 (55·0–69·0)	68·1 (61·6–76·1)	66·1 (58·6–73·5)	NA	63·0 (55·8–71·0)	68·2 (61·0–77·0)	68·9 (63·0–77·0)	66·0 (NA)	68·3 (NA)
	Sex												
		Male	190 (64%)	421 (69%)	189 (75%)	209 (66%)	226 (68%)	NA	177 (70%)	126 (70%)	221 (60%)	55 (65%)	189 (66%)
		Female	104 (35%)	179 (29%)	55 (22%)	108 (34%)	107 (32%)	NA	75 (30%)	55 (30%)	144 (40%)	29 (35%)	97 (34%)
		Unknown or other	2 (1%)	12 (2%)	9 (3%)	2 (1%)	1 (<1%)	NA	0	0	1 (<1%)	0	0
	Preoperative treatment status												
		Pretreated	0	0	117 (46%)	0	0	123 (5%)	0	49 (27%)	0	NA	0
		Not pretreated	418 (100%)	612 (100%)	136 (54%)	319 (100%)	334 (100%)	2516 (95%)	252 (100%)	132 (73%)	366 (100%)	NA	286 (100%)
	Laurén histological subtype												
		Intestinal	166 (56%)	219 (36%)	199 (79%)	206 (65%)	153 (46%)	1332 (50%)	111 (44%)	105 (58%)	221 (60%)	NA	153 (53%)‡
		Diffuse	74 (25%)	312 (51%)	45 (18%)	77 (24%)	61 (18%)	772 (29%)	132 (52%)	42 (23%)	89 (24%)	NA	NA‡
		Mixed or other	54 (18%)	69 (11%)	7 (3%)	35 (11%)	119 (36%)	258 (10%)	NA	34 (19%)	38 (10%)	NA	133 (47%)‡
		Unknown	2 (1%)	12 (2%)	2 (1%)	1 (<1%)	1 (<1%)	227 (9%)	9 (4%)	0	18 (5%)	NA	0
	UICC disease stage												
		Stage I	58 (20%)	1 (<1%)	NA	30 (9%)	41 (12%)	NA	0	32 (18%)	54 (15%)	NA	57 (20%)
		Stage II	66 (22%)	207 (34%)	NA	93 (29%)	104 (31%)	NA	97 (38%)	53 (29%)	77 (21%)	NA	76 (27%)
		Stage III	166 (56%)	392 (64%)	NA	190 (60%)	151 (45%)	NA	141 (56%)	72 (40%)	154 (42%)	NA	134 (47%)
		Stage IV	1 (<1%)	0	NA	4 (1%)	35 (10%)	NA	14 (6%)	20 (11%)	79 (22%)	NA	19 (7%)
		Unknown	5 (2%)	12 (2%)	NA	2 (1%)	3 (1%)	NA	0	4 (2%)	2 (1%)	NA	0
	Grade of differentiation												
		Grade 1	18 (6%)	NA	NA	17 (5%)	NA	NA	NA	9 (5%)	14 (4%)	NA	79 (28%)§
		Grade 2	76 (26%)	NA	NA	103 (32%)	NA	NA	NA	62 (34%)	114 (31%)	NA	..§
		Grade 3–4	200 (68%)	NA	NA	196 (61%)	NA	NA	NA	90 (50%)	200 (55%)	NA	206 (72%)
		Unknown	2 (1%)	NA	NA	3 (1%)	NA	NA	NA	20 (11%)	38 (10%)	NA	1 (<1%)
	Ground truth method												
		Microsatellite instability or mismatch repair deficient status	Immunohisto–chemistry	PCR	PCR	Immunohisto–chemistry	Genetic test	NA	Immunohisto–chemistry	Immunohisto–chemistry	PCR	Immunohisto–chemistry and PCR	PCR
		EBV status	EBER ISH	EBER ISH	NA	EBER ISH	Genetic test	NA	EBER ISH	EBER ISH	EBER ISH	EBER ISH	EBER ISH
		Digital slide scanner	3D Histech	Leica Aperio	Leica Aperio	Leica Aperio	Leica Aperio	NA	Leica Aperio	Roche Ventana	Leica Aperio	Hamamatsu	Leica Aperio

Data are n (%) or median (IQR), unless otherwise stated.. AUGSB=samples from University Hospital Augsberg, Germany. BERN=samples from University of Bern, Switzerland. CLASSIC=samples from the CLASSIC trial in South Korea. EBER=Epstein-Barr virus encoded small RNAs. EBV=Epstein-Barr virus. ISH=in-situ hybridisation. ITALIAN=samples from University of Siena, Italy. KCCH=samples from Kanagawa Cancer Center Hospital, Japan. KOELN=samples from University of Cologne, Germany. LEEDS=samples from Leeds Teaching Hospitals NHS Trust, UK. MAGIC=samples from the MAGIC trial in the UK. NA=not available. TCGA=samples from The Cancer Genome Atlas. TUM=samples from Technical University Munich, Germany. UICC=Union for International Cancer Control.

* Pooled cohort comprises BERN, CLASSIC, MAGIC, LEEDS, and TCGA cohorts.

† In the KOLEN cohort, EBV status was available for 89 patients but because only two were EBV positive we did not use data from this cohort for EBV status detection; therefore, the total number of patients included in this study from the KOLEN cohort was 84 (ie, those with microsatellite instability information available).

‡ Participants were divided into intestinal and non-intestinal in this cohort.

§ Grade 1 and 2 are pooled as “non-high grade” in this cohort.

Using within-cohort cross-validation in each of the ten cohorts, we found that microsatellite instability was detectable in nine of ten cohorts, with the lower bound of the 95% CI for the AUROC of the deep learning-based classifier above 0·5 (table 2). Among these nine cohorts, the AUROC ranged from 0·597 (95% CI 0·522–0·737) in the MAGIC cohort to 0·836 (0·795–0·880) in the TCGA cohort. In the KCCH cohort, microsatellite instability status was not detectable (AUROC 0·540 [0·477–0·592]; table 2). Data were available for detection of EBV status for all cohorts except MAGIC and KOELN. EBV status was detectable in five of these cohorts, with AUROC values ranging from 0·819 (0·752–0·841) in the TCGA cohort to 0·897 (0·513–0·966) in the TUM cohort (table 2). All possible sensitivity-specificity pairs for each cohort are visualised in the respective ROC curves in the appendix (p 12). Patient-level prediction scores differed significantly between patients with true microsatellite instability and microsatellite stability in seven of ten cohorts and between patients with true EBV positive and EBV negative status in six of eight cohorts (table 2). Variation of the number of bootstrapping experiments and the number of cross-validation folds did not affect the accuracy of detection (appendix pp 6–7).

**Table 2:** Performance of deep learning-based classifiers for detection of microsatellite instability and EBV

	**BERN**	**CLASSIC**	**MAGIC**	**LEEDS**	**TCGA**	Pooled cohort*	**KCCH**	**AUGSB**	**ITALIAN**	**KOELN**	**TUM**
**Performance for within-cohort cross-validation experiment**											
Microsatellite instability or mismatch repair deficient status	0·770 (0·718–0·882; p<0·0001)	0·744 (0·601–0·795; p<0·0001)	0·597 (0·522–0·737; p=0·052)	0·605 (0·529–0·656; p=0·010)	0·836 (0·795–0·880; p<0·0001)	NA	0·540 (0·477–0·592; p=0·46)	0·788 (0·645–0·885; p<0·0001)	0·785 (0·752–0·861; p<0·0001)	0·731 (0·642–0·802; p=0·47)	0·748 (0·683–0·796; p<0·0001)
EBV status	0·827 (0·650–0·924; p<0·0001)	0·864 (0·803–0·895; p<0·0001)	NA	0·842 (0·779–0·879; p<0·0001)	0·819 (0·752–0·841; p<0·0001)	NA	0·644 (0·494–0·814; p=0·026)	0·458 (0·305–0·608; p=0·65)	0·552 (0·350–0·782; p=0·48)	NA	0·897 (0·513–0·966; p<0·0001)
**Performance for external validation**											
Microsatellite instability or mismatch repair deficient status	..	..	..	..	..	0·761 (0·707–0·792; p<0·0001)	0·723 (0·676–0·794; p<0·0001)	0·758 (0·592–0·882; p=0·0004)	0·767 (0·726–0·830; p<0·0001)	0·863 (0·747–0·969; p=0·010)	0·793 (0·679–0·866; p<0·0001)
EBV status	..	..	..	..	..	0·810 (0·767–0·840; p<0·0001)	0·836 (0·653–0·966; p<0·0001)	0·672 (0·403–0·989; p=0·10)	0·859 (0·823–0·919; p<0·0001)	NA	0·676 (0·497–0·737; p=0·0002)

Data are AUROC (95% CI). AUGSB=samples from University Hospital Augsberg, Germany. AUROC=area under the receiver operating characteristic curve. BERN=samples from University of Bern, Switzerland. CLASSIC=samples from the CLASSIC trial in South Korea. EBV=Epstein-Barr virus. ITALIAN=samples from University of Siena, Italy. KCCH=samples from Kanagawa Cancer Center Hospital, Japan. KOELN=samples from University of Cologne, Germany. LEEDS=samples from Leeds Teaching Hospitals NHS Trust, UK. MAGIC=samples from the MAGIC trial in the UK. NA=not applicable. TCGA=samples from The Cancer Genome Atlas. TUM=samples from Technical University Munich, Germany.

* Pooled cohort comprises BERN, CLASSIC, MAGIC, LEEDS, and TCGA cohorts.

**Figure 1:**
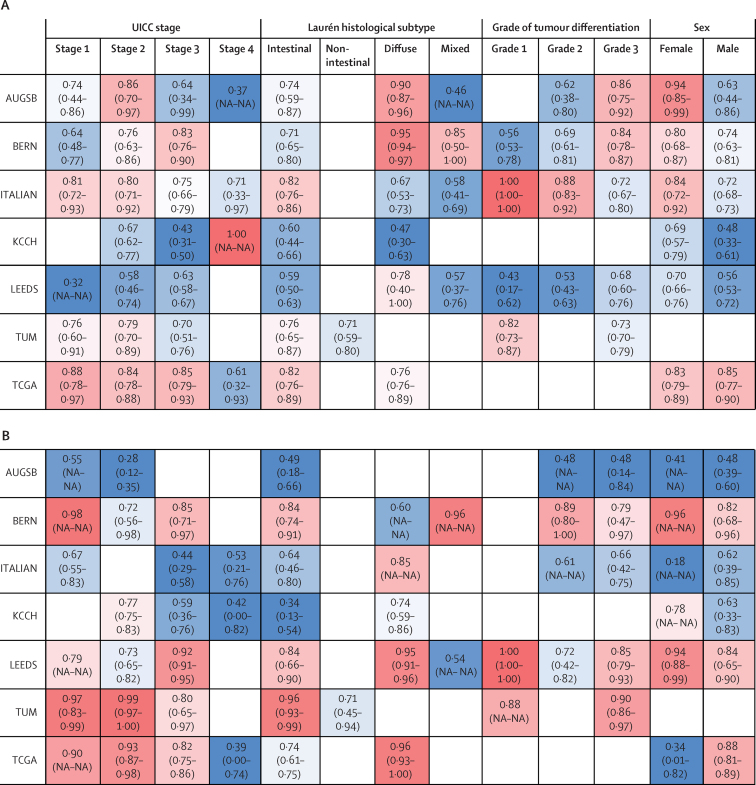
Subgroup-dependent performance of deep learning-based classifiers for detection of microsatellite instability and EBV Subgroup-dependent AUROCs for detection of microsatellite instability (A) and EBV (B). AUGSB=samples from University Hospital Augsberg, Germany. AUROC=area under the receiver operator curve. BERN=samples from University of Bern, Switzerland. CLASSIC=samples from the CLASSIC trial in South Korea. EBV=Epstein-Barr virus. ITALIAN=samples from University of Siena, Italy. KCCH=samples from Kanagawa Cancer Center Hospital, Japan. KOELN=samples from University of Cologne, Germany. LEEDS=samples from Leeds Teaching Hospitals NHS Trust, UK. MAGIC=samples from the MAGIC trial in the UK. NA=not available. TCGA=samples from The Cancer Genome Atlas. TUM=samples from Technical University Munich, Germany. UICC=Union for International Cancer Control.

For detection of microsatellite instability and EBV status, the performance of the classifier was usually lower in patients with UICC stage IV tumours than in other patients (figure 1). The performance of detection of microsatellite instability tended to be better among female than male patients (in five of six cohorts), whereas no consistent trend in performance by patient sex was observed for EBV detection. For EBV prediction, slightly higher AUROCs were achieved in diffuse-type than in intestinal-type gastric cancer, except for in the BERN and TUM cohorts (figure 1B). Although variations were observed from the general trends in subgroups with fewer than 50 patients, differences between cohorts were more pronounced than differences between subgroups (figure 1).

Re-training the microsatellite instability classifier on the combined training cohort using within-cohort three-fold cross-validation gave an AUROC of 0·761 (95% CI 0·707–0·792; table 2). When we re-trained the classifier on all patients in this training cohort and externally validated the classifier on each of the remaining five validation cohorts separately, microsatellite instability status was detectable from histology in all five cohorts, with AUROCs ranging from 0·723 (95% CI 0·676–0·794) for the KCCH cohort (for which microsatellite instability was undetectable via the previous within-cohort approach) to 0·863 (0·747–0·969) for the KOELN cohort (table 2). For EBV detection, a within-cohort experiment of the pooled training set gave an AUROC of 0·810 (0·767–0·840; table 2). Separate testing of the EBV classifier on each of the remaining four eligible cohorts yielded AUROCs between 0·672 (0·403–0·989) for the AUGSB cohort and 0·859 (0·823–0·919) for the ITALIAN cohort (table 2). The ITALIAN cohort consisted of tissue microarray samples containing a relatively small amount of tissue. EBV detection was unsuccessful in this cohort in the within-cohort experiment, with an AUROC of 0·552 (0·350–0·782; table 2); however, our external validation experiment resulted in an increase in the performance of EBV detection to an AUROC of 0·859 (0·823–0·919; table 2). Thus, EBV status was detectable in three of four validation cohorts (KCCH, ITALIAN, and TUM). For all cohorts, training on a pooled training dataset boosted performance of the classifier for detection of microsatellite instability and EBV status. AUROCs for external validation and corresponding highest predictive tiles are visualised in the appendix (p 13).

Training of a deep learning-based classifier to distinguish between patients with positive EBV status, those with microsatellite instability, and those with negative EBV and microsatellite instability status (double-negative gastric cancer) in a single classification step using within-cohort cross-validation was feasible in four of eight cohorts (CLASSIC, LEEDS, TCGA, and TUM) with lower 95% CI bounds that were higher than 0·5. Classification AUROCs in these four cohorts ranged from 0·694 (0·587–0·805) for the TUM cohort to 0·823 (0·767–0·850) for the LEEDS cohort across all classes (although both of these quoted AUROCs occurred for EBV detection; appendix p 8). In the CLASSIC cohort (the largest cohort), data were available for 36 patients with EBV-positive cancers, 30 patients with microsatellite instability, and 495 patients with double-negative cancers. In this cohort, positive EBV status was detected with an AUROC of 0·768 (0·750–0·801), microsatellite instability was detected with an AUROC of 0·795 (0·725–0·825), and double-negative status was detected with an AUROC of 0·819 (0·765–0·847). For the other cohorts (BERN, KCCH, AUGSB, and ITALIAN), the lower bound of the 95% CI was lower than 0·5 in at least one of the three classes. AUROCs for three-way classification are visualised in the appendix (p 14).

**Figure 2:**
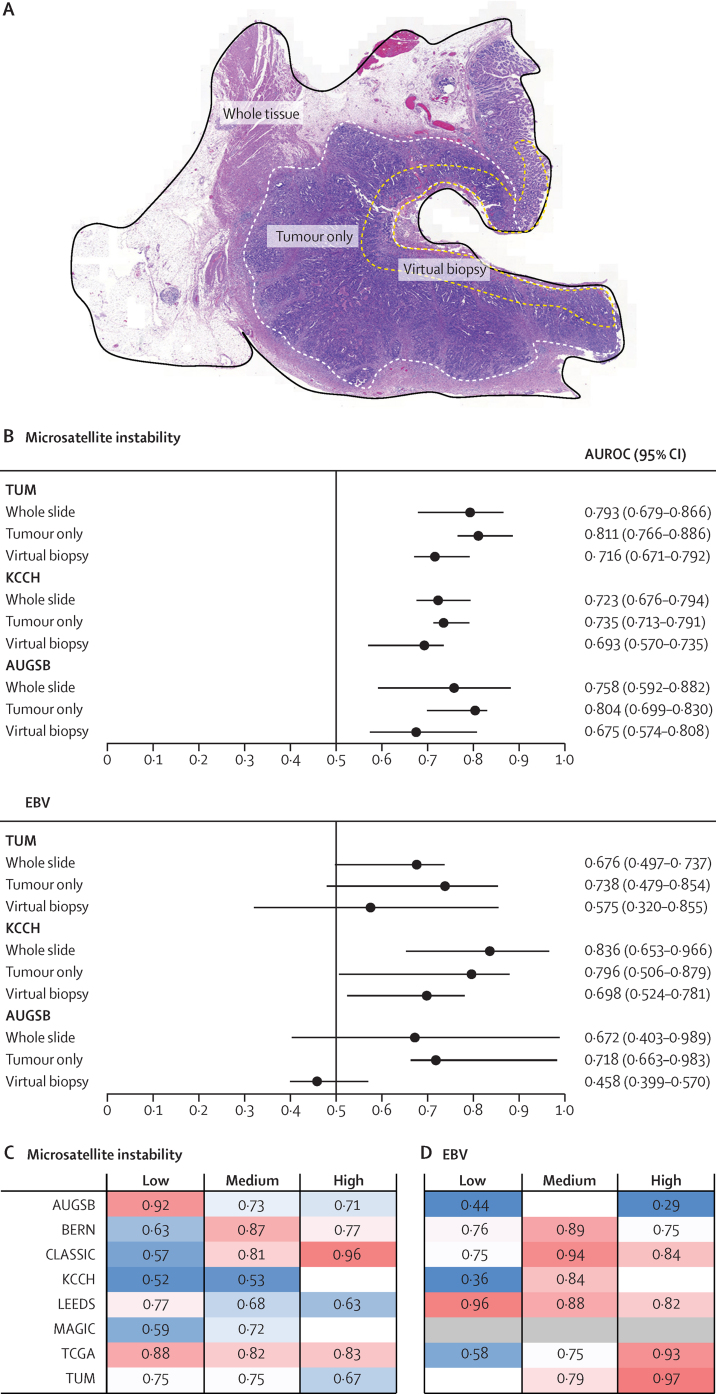
Effects of region-specific analysis and tumour-to-tissue ratio on classifier performance (A) Example tissue section, whole tumour annotation, and luminal surface annotation (ie, a virtual biopsy). (B) Microsatellite instability and EBV prediction scores for whole-slide images, tumour only, and virtual biopsy samples in the TUM, KCCH, and AUGSB cohorts. Prediction performance of the model for microsatellite instability (C) and EBV status (D) according to tumour-to-tissue ratio. Patients were stratified by the ratio between tumour-contianing area and total tissue area as follows: low was a tumour-to-tissue ratio of 0–0·33, medium was a ratio of 0·34–0·66, and high was a ratio of 0·66–1. AUROC=area under the receiver operating curve. AUGSB=samples from University Hospital Augsberg, Germany. BERN=samples from University of Bern, Switzerland. CLASSIC=samples from the CLASSIC trial in South Korea. EBV=Epstein-Barr virus. ITALIAN=samples from University of Siena, Italy. KCCH=samples from Kanagawa Cancer Center Hospital, Japan. LEEDS=samples from Leeds Teaching Hospitals NHS Trust, UK. MAGIC=samples from the MAGIC trial in the UK. TCGA=samples from The Cancer Genome Atlas. TUM=samples from Technical University Munich, Germany.

Among the KCCH, TUM, and AUGSB cohorts, the whole-slide image-based approach was marginally outperformed by the tumour-only approach for detection of microsatellite instability (figure 2; appendix p 9). For EBV detection, the tumour only-based approach had higher detection performance than the whole slide-based approach in the AUGSB cohort. EBV was not detectable via the whole-slide approach (AUROC 0·672 [95% CI 0·403–0·989]) because the lower 95% CI bound was below 0·5; however, the AUROC was increased to 0·718 (0·663–0·983) via the tumour-only approach, rendering EBV status detectable (figure 2B; appendix p 9). Prediction of microsatellite instability from virtual biopsies was less accurate than from whole-slide images but feasible in all three cohorts whereas EBV detection from virtual biopsies was only successful in the KCCH cohort (figure 2; appendix p 9).

No consistent trend in AUROCs was observed across the three strata of tumour-to-tissue ratios (high, medium, and low; figure 2C, D).

To identify specific predictive features from tiles with the highest prediction scores, we used the BERN cohort as an example because of its high performance for detection of microsatellite instability. The most highly predictive tiles for microsatellite instability contained tumour epithelium and lymphoid aggregates, whereas the highest scoring tiles for microsatellite stability contained both tumour and non-tumour tissue (appendix pp 13, 15). Among the highest predictive tiles for microsatellite instability, we identified tiles with activated lymphoid follicles. For EBV status, the most highly predictive tiles for EBV positivity contained mostly tumour tissue, whereas the tiles that were most highly predictive for EBV negativity contained both tumour and non-tumour tissue (appendix pp 13, 15). In whole-slide prediction heatmaps, highly predictive regions were mostly located in the tumour area (appendix p 13).

## Discussion

We assessed the performance of a deep learning-based classifier for the detection of microsatellite instability and EBV status in gastric cancer. While within-cohort cross-validation experiments resulted in pronounced performance differences between cohorts, external validation of a classifier that has been trained on a mixed training dataset significantly increased overall detection performance for both microsatellite instability and EBV status. Neither the investigated subgroups nor prespecified tumour-to-tissue ratios were significantly related with the detection performance of microsatellite instability or EBV status. Compared with non-annotated whole-slide images, detection of microsatellite instability or EBV status from annotated tumour regions did not improve classifier accuracy, whereas detection of microsatellite instability or EBV status from virtual biopsies resulted in reduced detection performance.

Deep learning has transformed digital pathology, enabling detection and subtyping of tumours.^13^, ^14^, ^15^ In gastric cancer, previous deep learning-based studies on molecular detection were limited to small datasets.^11^, ^21^, ^22^ However, adoption of deep learning-based biomarkers in clinical practice requires large-scale multicentre validation,^36^ which is especially relevant in the context of biases in AI systems.^37^ In our multicentre analysis across multiple countries, we found that pooling cohorts can improve performance, suggesting that a large and diverse dataset is important. Previously, a microsatellite instability classifier trained on the TCGA cohort, in which approximately 20% of patients are Asian, and tested on the KCCH cohort, in which 100% of patients are Asian, gave an AUROC of 0·69 (95% CI 0·52–0·82).^11^ When we trained our classifier on TCGA and four other cohorts with varying countries of origin, including another Asian cohort, prediction of microsatellite instability in the KCCH cohort yielded an AUROC of 0·723 (95% CI 0·676–0·794). More generally, we found that use of a classifier that was trained on a large multinational dataset outperformed classifiers trained in a within-cohort setup. We conclude that diverse training cohorts are necessary to obtain consistently high validation performance in gastric cancer.

Additionally, we analysed classification accuracy in clinical and pathological subgroups across our cohorts. None of the subgroups performed consistently better or worse than the overall cohort. Our finding that tumour annotations were not necessary to train a robust classifier and that robust classifiers can be trained even if all tiles from the whole-slide image are used raises questions about the relevance of extratumoural features such as peri-tumoural inflammatory cells or features in the adjacent non-neoplastic tissue for deep learning-based molecular detection. Generally, tumours with microsatellite instability or positive EBV status are known to influence the presence of immune cells in peritumoural and intratumoural tissue.^38^ Correspondingly, among the highest predictive tiles for microsatellite instability in the BERN cohort, we identified an activated lymphoid follicle in a tile highly predictive for microsatellite instability. We can infer that the presence of extratumoral tissue does not compromise the performance of digital detection of microsatellite instability or EBV, but its relevance—specifically the relevance of peritumoral lymphocytes—to the prediction needs to be further analysed.

Our study has several limitations. The relatively low absolute number of patients who were positive for features of interest proved to be a challenge for building a robust classifier in within-cohort experiments. Cohort-specific properties could add to this observation. For example, most of the digitised slides for the KCCH cohort had pen marks circling the tumour area. We expect these to have negatively affected our within-cohort accuracy. In the MAGIC cohort, almost 50% of the patients included had been pretreated with chemotherapy, which might have changed tumour morphology, negatively affecting the performance of the classifier. Finally, the AUGSB and ITALIAN cohorts both had a relatively low number of EBV positive tumours. Only three (2%) of 181 patients in the AUGSB cohort, and seven (2%) of 364 patients in the ITALIAN cohort were EBV positive. However, we found a solution for these problems: low classifier performance in the within-cohort experiments was overcome by training the classifier on a large multicentre cohort. A structural limitation to our analysis is the fact that the ground truth methods for microsatellite instability were developed in colorectal cancer, which could explain why microsatellite instability can be predicted in colorectal cancer with an even higher performance than we found here for gastric cancer.^16^ Our study shows that the applicability of a deep learning classifier can be increased by training on large and diverse cohorts. Still, gastric cancer seems to be an exceptionally difficult target for deep learning analysis and other issues still need to be addressed, such as the effect of pretreatment or ethnicity on performance.

For clinical adoption of deep learning, three steps are needed: proof of concept, large-scale validation, and regulatory approval.^36^ To our knowledge, this is the first large-scale validation study of any molecular deep learning-based biomarker in gastric cancer. Technical refinements with new architectures and training on even larger datasets could conceivably increase performance. Ultimately, deep learning-based analysis of haematoxylin and eosin-stained tissue genotyping could be used as a definitive test in gastric cancer because even imperfect predictors are useful as a pre-screening tool. By choosing a high-sensitivity operating point of moderate specificity, our test could pre-select patients for subsequent molecular testing.^11^ Pathology workflows across the world are predominantly based on glass slides. However, similar to the developments in radiology two decades ago, the digitisation of pathology is expected to happen within the foreseeable future.^36^, ^39^ Digital algorithms such as ours could potentially be added to such digital workflows, providing a fast and low-cost decision aid.

**This online publication has been corrected. The corrected version first appeared at thelancet.com/digital-health on August 19, 2021**

## Data sharing

All source codes to train and assess our deep learning classifiers are publicly available on GitHub. All images and patient data for the TCGA cohort are available online. All other data were provided by the respective study principal investigators and different data sharing policies apply as described previously in those original publications. We cannot make any individual patient-level data available to others, but these data can be requested from the respective pathology institutions as defined in the references for BERN,^23^ CLASSIC,^24^ MAGIC,^25^ LEEDS,^26^ KCCH,^26^ AUGSB,^4^ ITALIAN,^27^ KOELN,^28^ and TUM.^4^

## Declaration of interests

JNK declares consulting roles for OWKIN France and Panakeia (UK) without any direct connection to this work; these roles started in April, 2021, after conducting the present study. JNK also declares honoraria from MSD and Eisai. DC declares grants from Medimmune/AstraZeneca, Clovis, Eli Lilly, 4SC, Bayer, Celgene, Leap, and Roche, and Scientific Board Membership for OVIBIO. DJ declares consulting services and advisory board participation for CureVac AG, Definiens, F Hoffmann-La Roche, Genmab A-S, Life Science Inkubator GmbH, VAXIMM AG, OncoOne Research & Development Research GmbH, and Oncolytics Biotech; payment or honoraria from SKK Kliniken Heilbronn, Georg Thieme Verlag, Terrapinn, Touch Medical Medica, BMS GmbH & Co KG, and MSD; reimbursements for expert opinion on medical questions from Wilhelm-Sander Foundation, Else-Kröner-Fresenius Foundation, Scherer Foundation, and NordForsk; meeting support (ie, for travel) from Amgen, Oryx GmbH, Roche Glycart AG, Parexel.com, IKTZ HD GmbH, and BMS; and leadership in the BMS Foundation Immunooncology. All other authors declare no competing interests.
