# Correction to *Lancet Digit Health* 2021; published online Aug 17. https://doi.org/10.1016/S2589-7500(21)00133-3

**DOI:** 10.1016/S2589-7500(21)00206-5

**Published:** 2021-08-19

*Muti HS, Heij LR, Keller G, et al. Development and validation of deep learning classifiers to detect Epstein-Barr virus and microsatellite instability status in gastric cancer: a retrospective multicentre cohort study.* Lancet Digit Health *2021; published online Aug 17. https://doi.org/10.1016/S2589-7500(21)00133-3*—In figure 2A of this Article, the colours were missing. This correction has been made as of Aug 19, 2021.

