# Supplementary material for: Development and validation of deep learning classifiers to detect Epstein-Barr virus and microsatellite instability status in gastric cancer: a retrospective multicentre cohort study
Source: Lancet Digit Health. 2021 Aug 17;3(10):e654–64. doi: 10.1016/S2589-7500(21)00133-3 (PMC8460994; doi:10.1016/S2589-7500(21)00133-3)
Supplement: Supplementary appendix [file mmc1.pdf]

THE LANCET
Digital Health

Supplementary appendix

This appendix formed part of the original submission and has been peer reviewed. We post it as supplied by the authors.

Supplement to: Muti HS, Heij LR, Keller G, et al. Development and validation of deep learning classifiers to detect Epstein-Barr virus and microsatellite instability status in gastric cancer: a retrospective multicentre cohort study. *Lancet Digit Health* 2021; published online Aug 17. https://doi.org/10.1016/S2589-7500(21)00133-3.

Appendix

STARD Guidelines 2

Training Hyperparameters 5

Repetition of experiment with 1000-fold bootstrapping 6

Relationship between cross validation folds and performance 7

Three-way classification results........ 8

Raw data for Figure 2 (Regional analysis)........ 9

Consort Diagrams of patient flow 10

Study design........ 11

AUROC curves for the internal validation experiment........ 12

AUROC curves and highest predictive tiles for the external validation experiment 13

Three-way classification AUROC curves........ 14

Detailed feature visualization 15

Wholeslide Prediction Heatmaps 16

STARD Guidelines

Section & Topic	No	Item	Reported on page #
TITLE OR ABSTRACT			
	1	Identification as a study of diagnostic accuracy using at least one measure of accuracy (such as sensitivity, specificity, predictive values, or AUC)	1
ABSTRACT			
	2	Structured summary of study design, methods, results, and conclusions (for specific guidance, see STARD for Abstracts)	2
INTRODUCTION			
	3	Scientific and clinical background, including the intended use and clinical role of the index test	5
	4	Study objectives and hypotheses	5
METHODS			
Study design	5	Whether data collection was planned before the index test and reference standard were performed (prospective study) or after (retrospective study)	5
Participants	6	Eligibility criteria	6
	7	On what basis potentially eligible participants were identified (such as symptoms, results from previous tests, inclusion in registry)	6
	8	Where and when potentially eligible participants were identified (setting, location and dates)	6 (see original publications)
	9	Whether participants formed a consecutive, random or convenience series	6 (see original publications)
Test methods	10a	Index test, in sufficient detail to allow replication	7-8, 20-21
	10b	Reference standard, in sufficient detail to allow replication	7-8

	11	Rationale for choosing the reference standard (if alternatives exist)	not applicable
	12a	Definition of and rationale for test positivity cut-offs or result categories of the index test, distinguishing pre-specified from exploratory	not applicable (AUC is independent from cut-offs)
	12b	Definition of and rationale for test positivity cut-offs or result categories of the reference standard, distinguishing pre-specified from exploratory	not applicable (AUC is independent from cut-offs)
	13a	Whether clinical information and reference standard results were available to the performers/readers of the index test	7-8
	13b	Whether clinical information and index test results were available to the assessors of the reference standard	6 (see original publications)
Analysis	14	Methods for estimating or comparing measures of diagnostic accuracy	8
	15	How indeterminate index test or reference standard results were handled	not applicable
	16	How missing data on the index test and reference standard were handled	6
	17	Any analyses of variability in diagnostic accuracy, distinguishing pre-specified from exploratory	7-8
	18	Intended sample size and how it was determined	6 (see original publications)
RESULTS			
Participants	19	Flow of participants, using a diagram	6 (reference to Suppl. Figure 1)
	20	Baseline demographic and clinical characteristics of participants	6 (reference to Table 2)
	21a	Distribution of severity of disease in those with the target condition	6 (reference to Table 2)

	21b	Distribution of alternative diagnoses in those without the target condition	not applicable (patients without target condition are excluded)
	22	Time interval and any clinical interventions between index test and reference standard	6 (see original publications)
Test results	23	Cross tabulation of the index test results (or their distribution)<br>by the results of the reference standard	8 (reference to Table 1)
	24	Estimates of diagnostic accuracy and their precision (such as 95% confidence intervals)	8-10
	25	Any adverse events from performing the index test or the reference standard	not applicable (analysis was performed on digitized whole slide images)
DISCUSSION			
	26	Study limitations, including sources of potential bias, statistical uncertainty, and generalisability	11-12
	27	Implications for practice, including the intended use and clinical role of the index test	12
OTHER INFORMATION			
	28	Registration number and name of registry	N/A
	29	Where the full study protocol can be accessed	N/A
	30	Sources of funding and other support; role of funders	20

Suppl. Table 1: STARD checklist for the present study. N/A means not applicable.

Training Hyperparameters

Description	Parameter name	Value
Upper limit of tiles per patient, single cohort	MaxBlockNum	2000
Upper limit of tiles per patient, merged cohorts	MaxBlockNum	1000
Trainable layers (hot layers)	hotLayers	30
Number of epochs	MaxEpochs	8
Mini batch size	MiniBatchSize	512
Initial learning rate	InitialLearnRate	0.00005
L2 Regularization	L2Regularization	0.0001

Suppl. Table 2: Hyperparameters for the Deep Learning system.

Repetition of experiment with 1000-fold bootstrapping

	BERN	CLASS	MAGIC	LEEDS	TCGA	KCCH	AUGSB	ITALIAN	KOELN	TUM
**Performance for within-cohort experiments (cross-validation)**										
**AUROC** MSI/dMMR xval	**0.770** [0.708; 0.832]	**0.744** [0.66; 0.829]	**0.597** [0.475; 0.718]	**0.605** [0.512; 0.695]	**0.836** [0.783; 0.890]	**0.54** [0.432; 0.645]	**0.788** [0.684; 0.886]	**0.785** [0.722; 0.845]	**0.731** [0.627; 0.835]	**0.748** [0.669; 0.820]
**AUROC** EBV xval	**0.827** [0.692; 0.947]	**0.864** [0.803; 0.913]	**N/A**	**0.842** [0.751; 0.916]	**0.819** [0.731; 0.895]	**0.644** [0.439; 0.812]	**0.458** [0.207; 0.767]	**0.552** [0.357; 0.749]	**N/A**	**0.897** [0.782; 0.983]
**Performance for external validation (train on five cohorts [pooled], test on five cohorts [separately])**										
**AUROC** MSI/dMMR test	**0.745** [0.708; 0.780]					**0.723** [0.615; 0.824]	**0.758** [0.635; 0.861]	**0.767** [0.711; 0.825]	**0.862** [0.767; 0.963]	**0.793** [0.722; 0.861]
**AUROC** EBV test	**0.810** [0.764; 0.859]					**0.836** [0.692; 0.950]	**0.672** [0.405; 0.983]	**0.859** [0.776; 0.940]	**N/A**	**0.676** [0.433; 0.932]

Suppl. Table 3: Reproduction of the main experimental results with 1000-fold bootstrapping and a different programming environment. This table reports the same results as Table 1 but with 1000-fold bootstrapped 95% confidence intervals which were calculated with Python/sklearn.

Relationship between cross validation folds and performance

	AUROC fold 1	AUROC fold 2	AUROC fold 3	AUROC fold 4	AUROC fold 5	AUROC fold 6	AUROC fold 7	AUROC fold 8	AUROC fold 9	AUROC MOF	AUROC concat
**2-fold cross-val**	0.75265	0.74906	N/A	N/A	N/A	N/A	N/A	N/A	N/A	0.750855	**0.74055**
**3-fold cross-val**	0.78954	0.6858	0.82313	N/A	N/A	N/A	N/A	N/A	N/A	0.766157	**0.75529**
**4-fold cross-val**	0.79048	0.76032	0.62482	0.82612	N/A	N/A	N/A	N/A	N/A	0.750435	**0.71334**
**5-fold cross-val**	0.79289	0.58946	0.66	0.87075	0.81699	N/A	N/A	N/A	N/A	0.746018	**0.72373**
**6-fold cross-val**	0.88605	0.64456	0.83503	0.7415	0.70578	0.84014	N/A	N/A	N/A	0.77551	**0.74183**
**7-fold cross-val**	0.62731	0.8912	0.75694	0.8588	0.78472	0.85648	0.85648	N/A	N/A	0.804561	**0.76904**
**8-fold cross-val**	0.78125	0.92258	0.57527	0.75521	0.85484	0.9375	0.80323	0.8151	N/A	0.805623	**0.75477**
**9-fold cross-val**	0.76071	0.95357	0.77857	0.71875	0.66964	0.75893	0.95	0.81071	0.775	0.79732	**0.78028**

Suppl. Table 4: Relationship between number of cross validation folds with classifier performance. In the BERN cohort, a classifier was trained to predict MSI status in a within-cohort experiment, mirroring experiment #1. To demonstrate the robustness of the performance with respect to the number of cross validation folds, the same experiment was repeated for 2, 3, 4, 5, 6, 7, 8 and 9 fold cross-validation. Only 200 tiles were used per patient, otherwise the same hyperparameters as in experiment #1 were used. MOF = mean of folds, concat. = concatenation of patient predictions before calculating AUROC.

Three-way classification results

	BERN	CLASS	MAGIC	LEEDS	TCGA	KCCH	AUGSB	ITALIAN	KOELN	TUM
N EBV+ MSI+ neg	8+ 42+ 224	36+ 30+ 495	N/A	13+ 30+ 253	27+ 58+ 248	11+ 22+ 200	3+ 16+ 162	5+ 68+ 213	N/A	8+ 24+ 233
mean AUROC EBV xval	**0.717** [0.447, 0.818]	**0.768** [0.750, 0.801]	N/A	**0.823** [0.767, 0.850]	**0.815** [0.789, 0.872]	**0.624** [0.362, 0.843]	**0.423** [0.258, 0.538]	**0.457** [0.454, 0.568]	N/A	**0.694** [0.587, 0.805]
mean AUROC MSI xval	**0.760** [0.715, 0.792]	**0.795** [0.725, 0.825]	N/A	**0.713** [0.567, 0.798]	**0.803** [0.718, 0.824]	**0.522** [0.338, 0.688]	**0.688** [0.598, 0.755]	**0.618** [0.557, 0.656]	N/A	**0.738** [0.674, 0.808]
mean AUROC neg xval	**0.753** [0.707, 0.851	**0.819** [0.765, 0.847]	N/A	**0.762** [0.681, 0.827]	**0.794** [0.765, 0.844]	**0.644** [0.588, 0.698]	**0.553** [0.553, 0.745]	**0.631** [0.595, 0.695]	N/A	**0.786** [0.684, 0.863]

Suppl. Table 5: Three-way-classifier for EBV-positive, MSI and double-negative tumors. *Three-fold cross validated within-cohort experiment for each cohort. Neg: double negative cases. N: number of patients (cases).*

Raw data for Figure 2 (Regional analysis)

	TUM whole slide*	TUM tumor only	TUM luminal only	KCCH whole slide*	KCCH tumor only	KCCH luminal only	AUGSB whole slide*	AUGSB tumor only	AUGSB luminal only
N MSI+ MSS =total	34 + 241 = 275	33 + 237 = 270	33 + 232 = 265	22 + 213 = 235	22 + 210 = 222	22 + 194 = 216	16 + 165 = 181	16 + 164 = 180	16 + 164 = 180
N EBV pos+neg = total	8 + 267 = 275	8 + 262 = 270	8 + 257 = 265	11 + 223 = 234	11 + 217 = 228	10 + 206 = 216	3 + 178 = 181	3 + 177 = 180	3 + 177 = 180
AUROC MSI/dMMR test	**0.793** [0.679, 0.866]	**0.811** [0.766, 0.886]	**0.716** [0.671, 0.792]	**0.723** [0.676, 0.794]	**0.735** [0.713, 0.791]	**0.693** [0.570, 0.735]	**0.758** [0.592, 0.882]	**0.804** [0.699, 0.830]	**0.675** [0.574, 0.808]
AUROC EBV test	**0.676** [0.497, 0.737]	**0.738** [0.479, 0.854]	**0.575** [0.320, 0.855]	**0.836** [0.653, 0.966]	**0.796** [0.506, 0.879]	**0.698** [0.524, 0.781]	**0.672** [0.403, 0.989]	**0.718** [0.663, 0.983]	**0.458** [0.399, 0.570]

Suppl. Table 6: Deployment of multi-cohort classifiers (trained on BERN, CLASSIC, MAGIC, LEEDS, TCGA) to whole slide, tumor only and luminal region in the validation cohort. (* same as Table 2). This is the raw data for Figure 4.

Consort Diagrams of patient flow

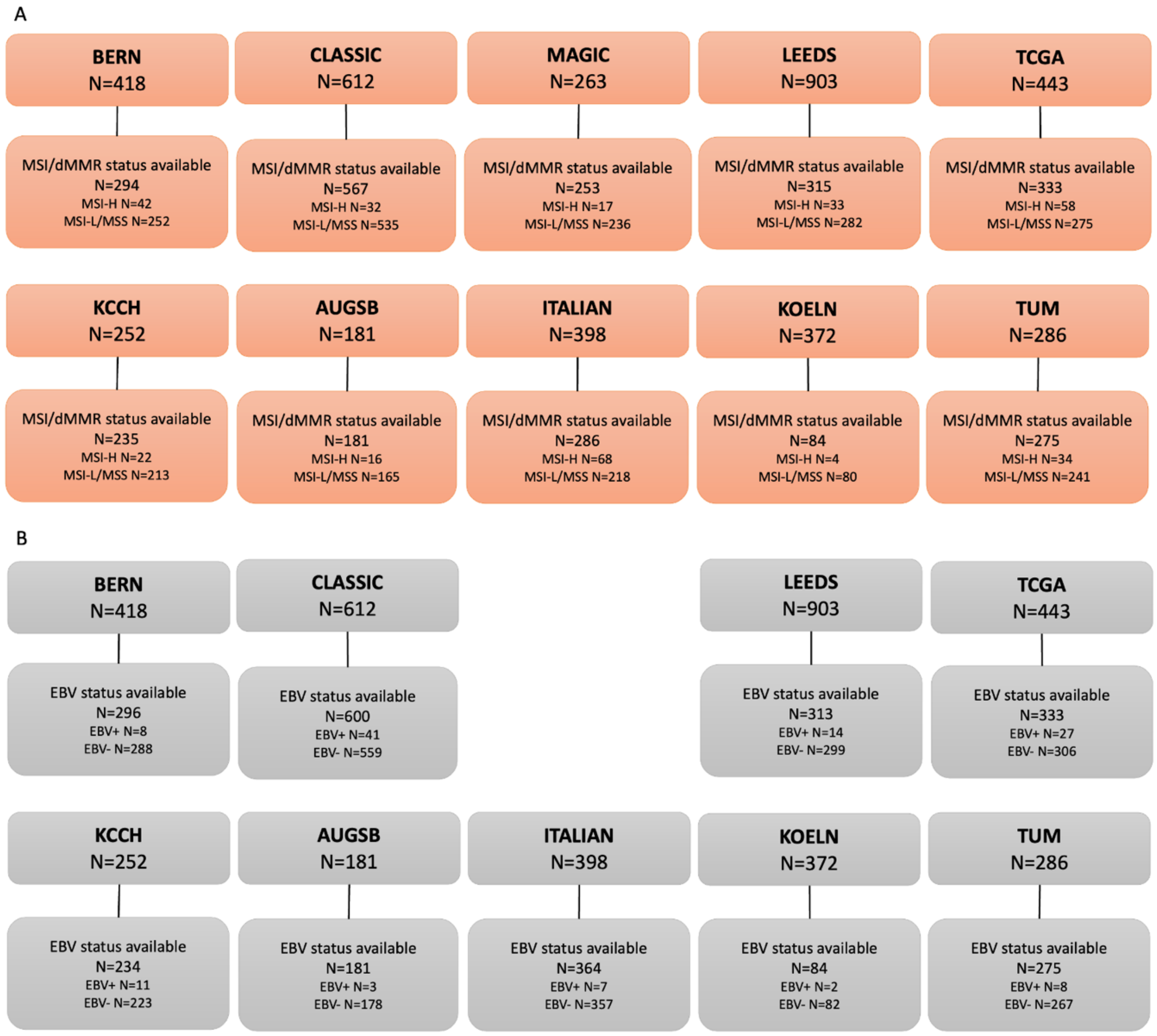

Suppl. Figure 1: Cohort-wise consort diagrams depicting the flow of patients for each cohort. (A) Consort diagrams for MSI/dMMR status prediction experiments for all cohorts. (B) Consort diagrams for EBV status prediction experiments for all cohorts. MSI/dMMR: microsatellite instability or mismatch repair deficiency; MSI-H: high microsatellite instability; MSI-L: low microsatellite instability; MSS: microsatellite stability; EBV+: Epstein-Barr-Virus positive; EBV-: Epstein-Barr-Virus negative.

Study design

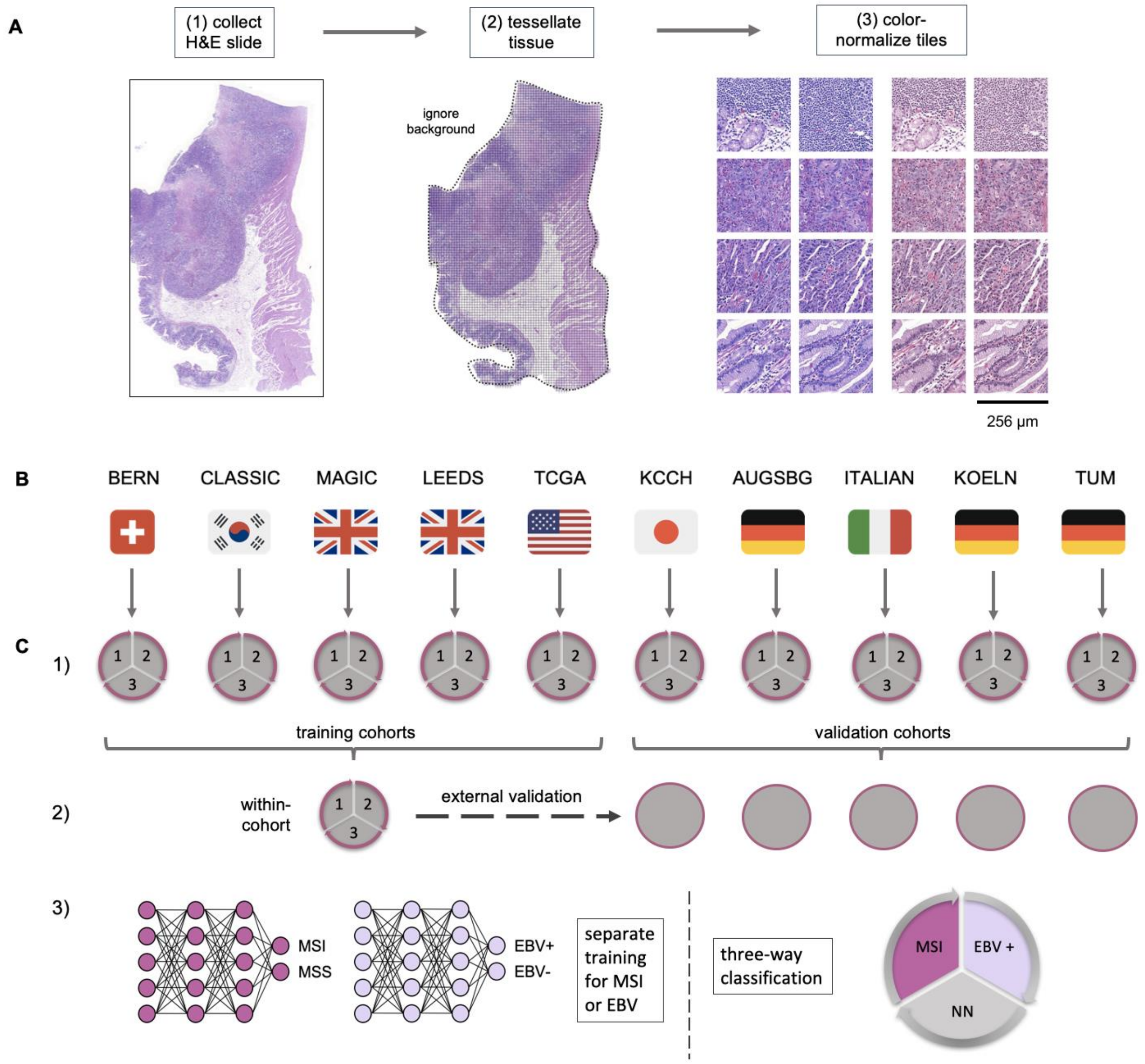

Suppl. Figure 2: Study outline. (A) A whole slide image containing a range of tissue types (1) is automatically tessellated without manual tumor annotations (2) and the resulting tiles are color-normalized (3). (B) Our large and diverse dataset consists of ten gastric cancer cohorts from seven countries. (C) Deep neural networks were trained and performance was assessed by internal cross-validation in each cohort (1) and by training on a pooled cohort and validating on the remaining cohorts (2) for MSI and EBV separately (3), compared to a three-way-classifier, where all parameters are assessed at once (3). H&E: hematoxylin and eosin; MSI: microsatellite instability; MSS: microsatellite stability; EBV: Epstein-Barr Virus; EBV+: EBV positive, EBV-: EBV negative, NN: double negative. Image credit for flags: Twitter Twemoji (CC-BY license).

AUROC curves for the internal validation experiment

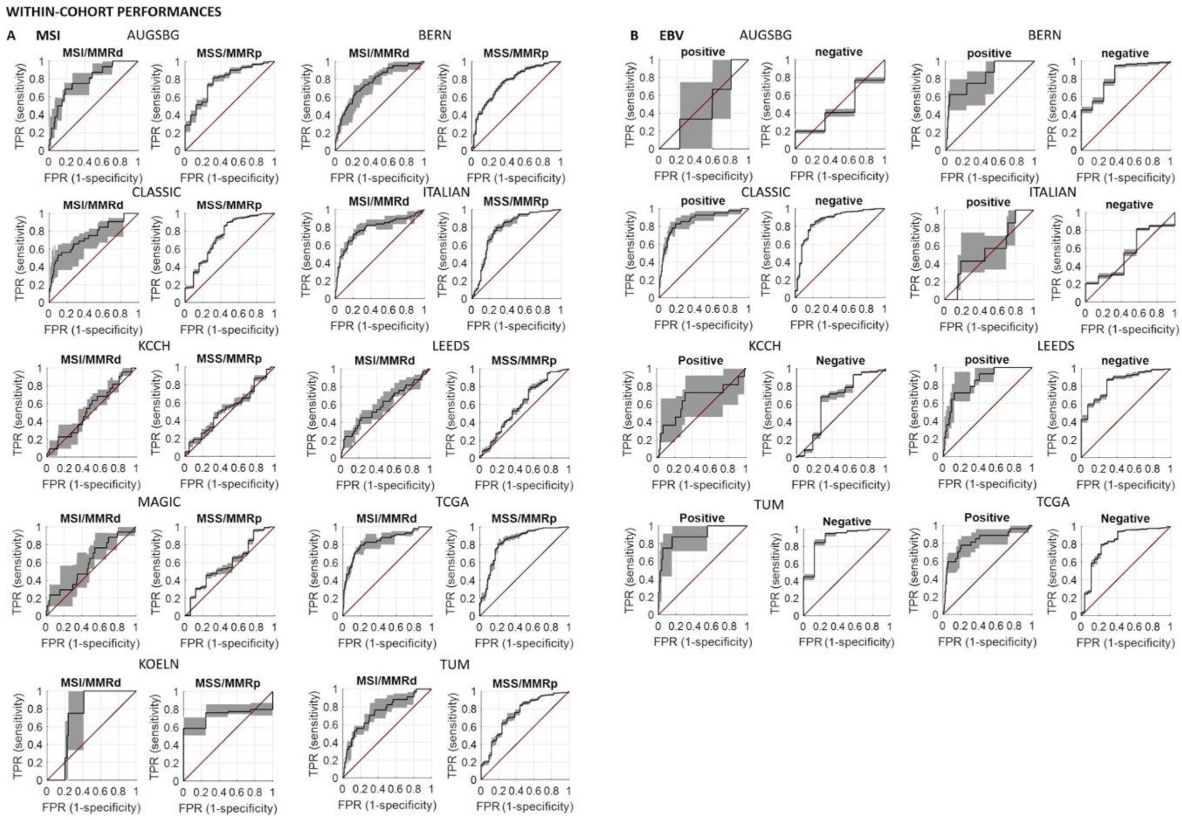

Suppl. Figure 3: Area under the Receiver Operating Curve graphics for MSI/dMMR and EBV prediction. (A) AUROCs for MSI/dMMR prediction in the within-cohort internal validation experiment (experiment #1). (B) AUROCs for EBV prediction in the within-cohort internal validation experiment (experiment #1). AUROCs: Area under the receiver operating curves; MSI/dMMR: microsatellite instability or mismatch repair deficiency; EBV: Epstein-Barr-Virus.

AUROC curves and highest predictive tiles for the external validation experiment

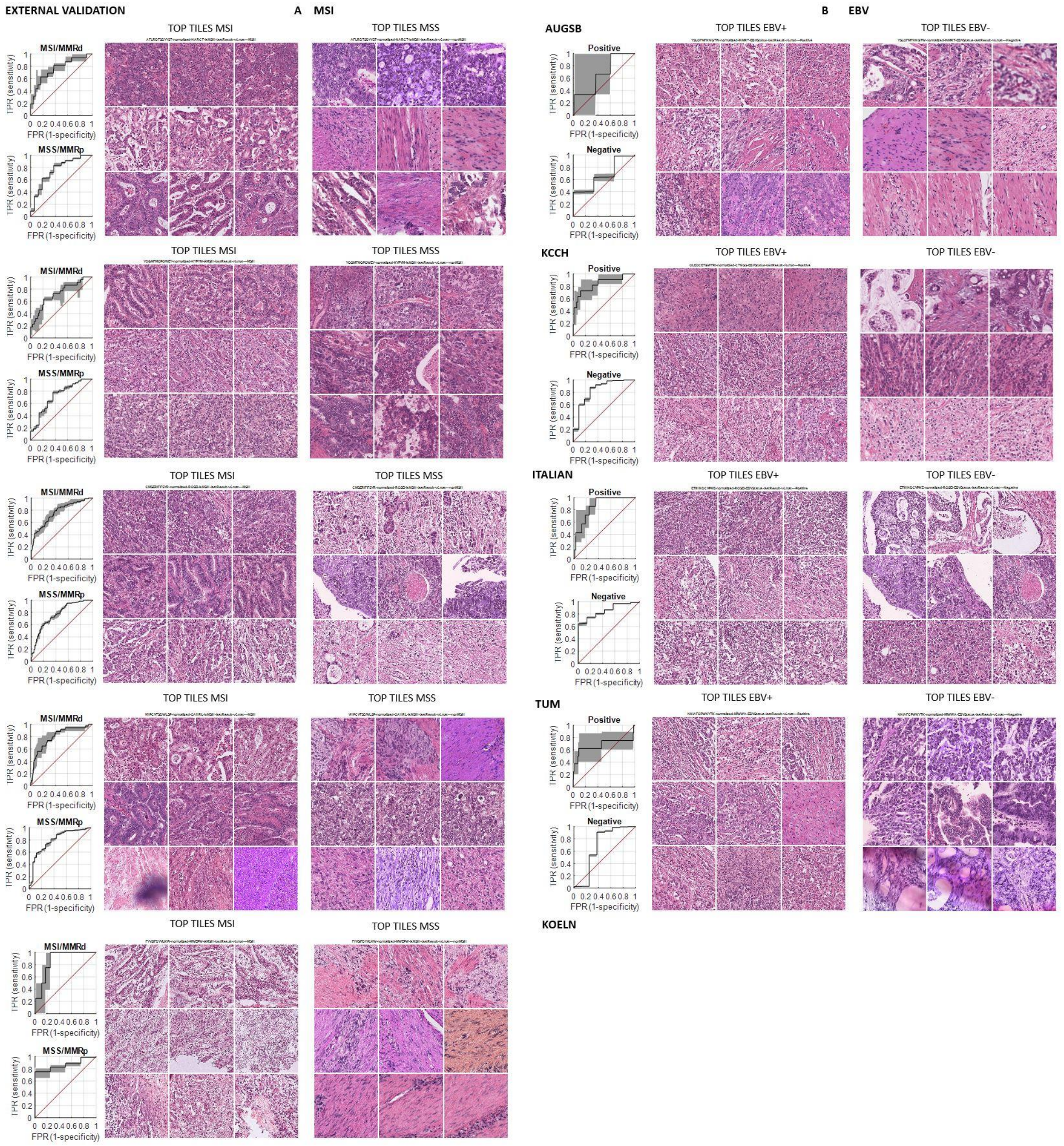

Suppl. Figure 4: AUROCs and corresponding highest predictive tiles of the external validation experiment. (A) AUROCs and corresponding three highest predictive tiles from the three highest predictive patients for MSI/dMMR prediction per validation cohort. (B) AUROCs and corresponding three highest predictive tiles from the three highest predictive patients for EBV prediction per validation cohort. KOELN did not include enough EBV positive patients to generate a prediction. AUROCs: Area under the receiver operating curves; MSI/dMMR: microsatellite instability or mismatch repair deficiency; EBV: Epstein-Barr-Virus.

Three-way classification AUROC curves

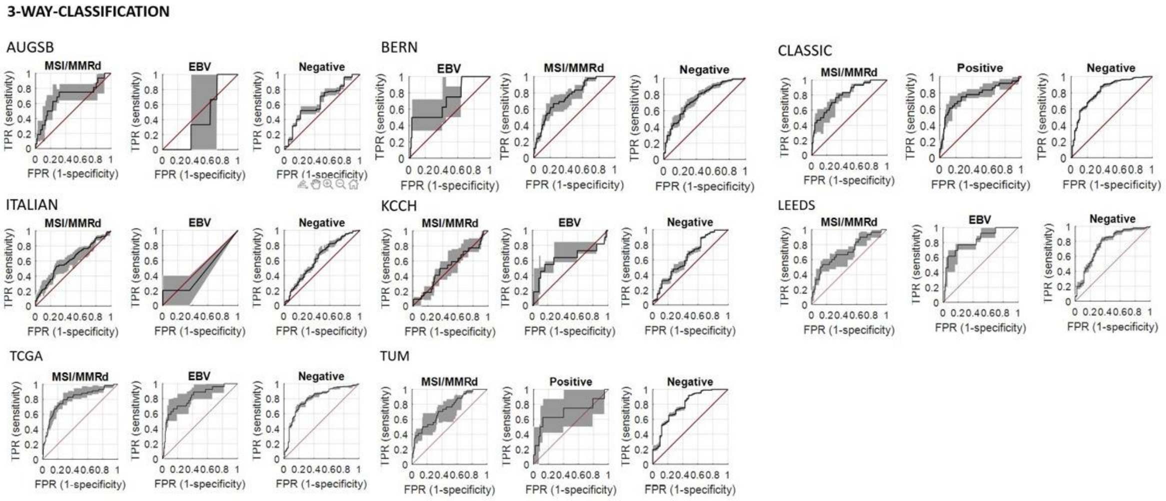

Suppl. Figure 5: Area under the Receiver Operating Curve graphics for three-way-classification. AUROC values for each cohort for MSI/dMMR, EBV positivity and double negativity resulting from a three-way-classifier generated in a within-cohort cross-validated design. MAGIC and KOELN had to be excluded from this experiment because there were not enough EBV positive cases in these cohorts. AUROC: Area under the receiver operating curve; MSI/dMMR: microsatellite instability or mismatch repair deficiency.

Detailed feature visualization

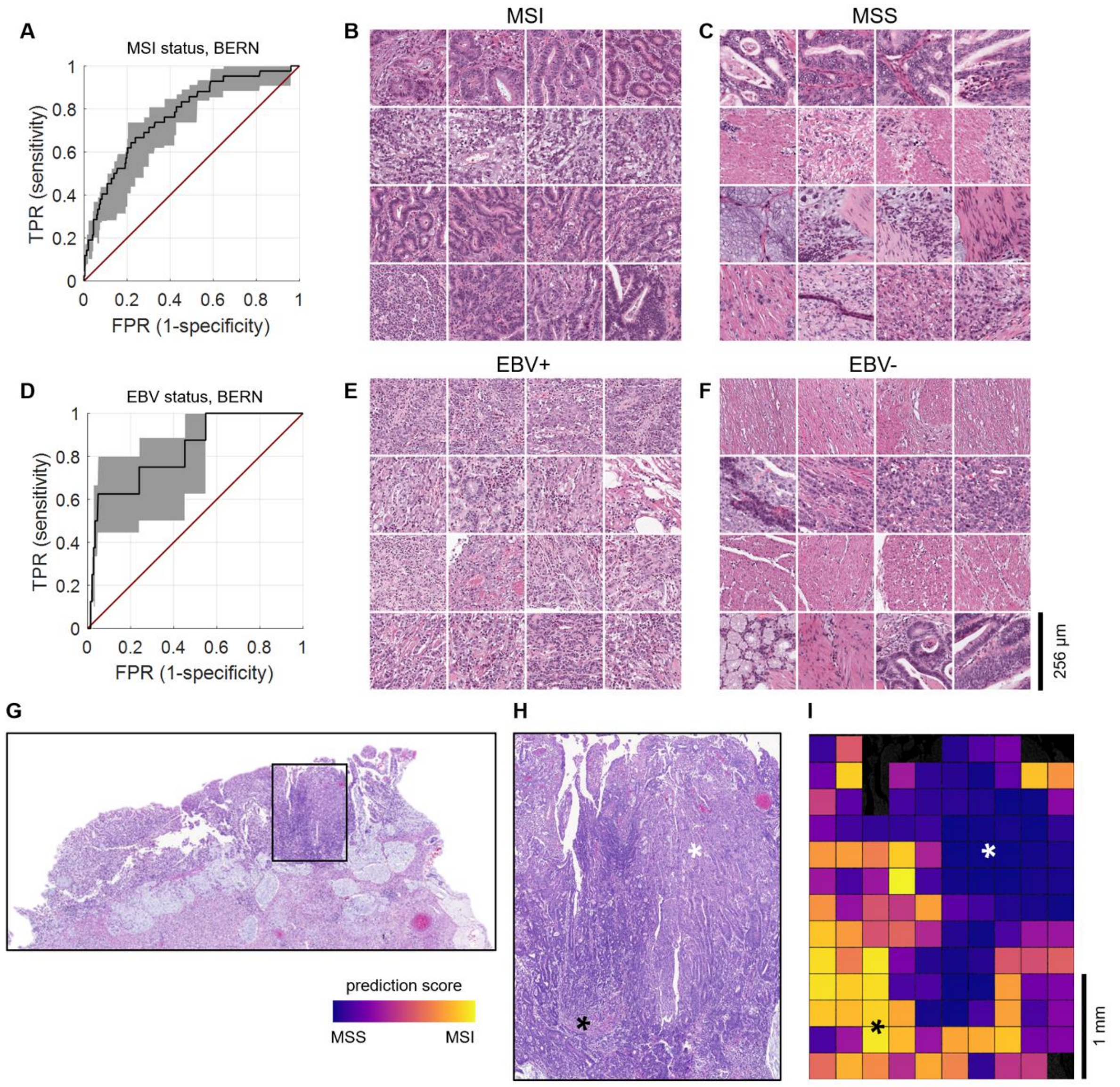

Suppl. Figure 6: Detailed feature visualization. (A) Receiver operating curve for MSI/dMMR classifier trained and tested on the BERN cohort. (B) Highest predictive tiles for microsatellite instability. (C) Highest predictive tiles for microsatellite stability. (D) Receiver operating curve for EBV classifier trained and tested on the BERN cohort. (E) Highest predictive tiles for EBV positivity. (F) Highest predictive tiles for EBV negativity. (G) Slide overview of an example image from the BERN cohort. (H) Enlarged detail: tumor area (black star) and non-tumorous gastric mucosa (white star). (I) Corresponding prediction map for MSI status. MSI: Microsatellite instability, MSS: Microsatellite stability, EBV: Epstein-Barr Virus.

Wholeslide Prediction Heatmaps

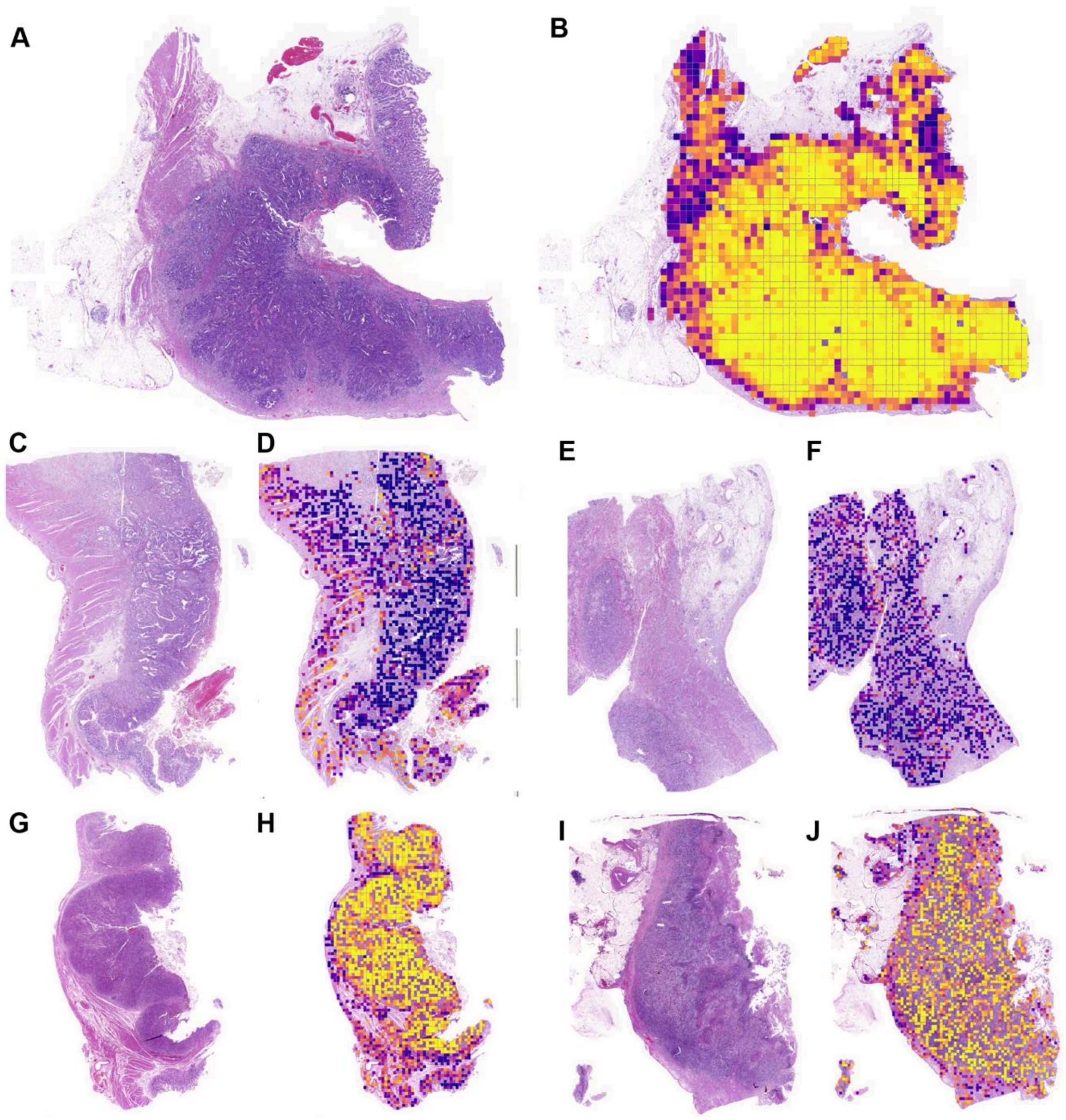

Suppl. Figure 8: Whole slide images and corresponding MSI prediction maps from the BERN cohort. (A) and (B), (C) and (D), (E) and (F), (G) and (H) and (I) and (J) are corresponding image-map pairs for representative patients. The color scale in all panels is identical to Figure 3E and ranges from blue (predicted non-MSI) to yellow (predicted MSI). Note that the number of tiles per patient was limited to 2000 during training, which is why some heatmaps do not cover the entire tissue area.
